# Development and validation of a hybrid deep learning–machine learning approach for severity assessment of COVID-19 and other pneumonias

**DOI:** 10.1038/s41598-023-40506-w

**Published:** 2023-08-17

**Authors:** Doohyun Park, Ryoungwoo Jang, Myung Jin Chung, Hyun Joon An, Seongwon Bak, Euijoon Choi, Dosik Hwang

**Affiliations:** 1 School of Electrical and Electronic Engineering, Yonsei University, Seoul, Republic of Korea; 2 Vuno Inc., Seoul, Republic of Korea; 3 Medical AI Research Center, Samsung Medical Center, Seoul, 06351 Republic of Korea; 4 Department of Radiology, Samsung Medical Center, Sungkyunkwan University School of Medicine, Seoul, 06351 Republic of Korea; 5 Department of Artificial Intelligence, Yonsei University, Seoul, Republic of Korea; 6 Center for Healthcare Robotics, Korea Institute of Science and Technology, 5, Hwarang-ro 14-gil, Seongbuk-gu, Seoul, 02792 Republic of Korea; 7 Department of Oral and Maxillofacial Radiology, Yonsei University College of Dentistry, Seoul, Republic of Korea; 8 Department of Radiology and Center for Clinical Imaging Data Science (CCIDS), Yonsei University College of Medicine, Seoul, Republic of Korea

**Keywords:** Infectious diseases, Diagnosis, Medical imaging

## Abstract

The Coronavirus Disease 2019 (COVID-19) is transitioning into the endemic phase. Nonetheless, it is crucial to remain mindful that pandemics related to infectious respiratory diseases (IRDs) can emerge unpredictably. Therefore, we aimed to develop and validate a severity assessment model for IRDs, including COVID-19, influenza, and novel influenza, using CT images on a multi-centre data set. Of the 805 COVID-19 patients collected from a single centre, 649 were used for training and 156 were used for internal validation (D1). Additionally, three external validation sets were obtained from 7 cohorts: 1138 patients with COVID-19 (D2), and 233 patients with influenza and novel influenza (D3). A hybrid model, referred to as Hybrid-DDM, was constructed by combining two deep learning models and a machine learning model. Across datasets D1, D2, and D3, the Hybrid-DDM exhibited significantly improved performance compared to the baseline model. The areas under the receiver operating curves (AUCs) were 0.830 versus 0.767 (*p* = 0.036) in D1, 0.801 versus 0.753 (*p* < 0.001) in D2, and 0.774 versus 0.668 (*p* < 0.001) in D3. This study indicates that the Hybrid-DDM model, trained using COVID-19 patient data, is effective and can also be applicable to patients with other types of viral pneumonia.

## Introduction

Since the outbreak of coronavirus disease 2019 (COVID-19) in 2019, the pandemic has had a profound and widespread impact, resulting in a significant increase in mortality globally. Particularly, elderly individuals and those have severe underlying medical conditions are at a higher risk of experiencing severe complications^1–5^. This alarming context highlighted the pressing need for a robust severity assessment system to guarantee appropriate care for severe patients. Fortuitously, as of now, COVID-19 is transitioning into the endemic phase. Nonetheless, it is crucial to remain mindful that pandemics related to infectious respiratory diseases (IRDs) can emerge unpredictably. This reality underscores the importance of not only managing the current situation but also preparing for other IRDs such as viral pneumonia (VP) and bacterial pneumonia (BP) to be better equipped for potential future outbreaks.

The presence of multifocal ground-glass opacity (GGO), consolidation, reticular opacity, and crazy-paving pattern in the lung fields is frequently observed in patients diagnosed with pneumonia, with GGO and consolidation being the most prevalent findings^6–9^. As evidenced by Park et al., the diagnostic performance for patients can be enhanced by taking various characteristics of lung abnormalities, such as GGO and consolidation, into consideration^10^. In effect, COVID-19 severity assessment algorithms primarily focus on pulmonary involvement when utilizing computed tomography (CT) scans, as the severity of the disease in patients with COVID-19 can be determined by analyzing the extent of lung involvement^11^. Furthermore, numerous studies have reported a correlation between the quantitative measurement of lung involvement in CT scans and laboratory findings as well as clinical parameters, often using machine learning (ML) techniques or a stratified scoring system for the analysis^12–14^. For instance, Lessmann et al. reported that the severity of COVID-19 patients can be determined from CT images by calculating the percentage of affected lung tissue per lobe^15^. Wenli et al. demonstrated that texture features for lesion volume and non-lesion lung volume are instrumental in determining the severity of COVID-19^16^. In this study, we limit our focus to the most common patterns of lesions, GGO and consolidation, as previously reported in relevant literature.

To date, several studies have utilized deep learning (DL) for the severity assessment of COVID-19 patients. Zhang et al. demonstrated the application of two imaging biomarkers derived from lung field and lung abnormality segmentation models for the severity assessment of COVID-19 patients^17^. Similarly, Goncharov et al. leveraged DL to generate a segmentation mask and calculate the affected lung percentage for severity assessment purposes^18^. Chieregato et al. presented a method that combined laboratory and clinical data with imaging features^19^. They extracted imaging features from CT scans using a DL model and integrated them into a CatBoost ML model, along with tabular data. Gao et al. proposed a dual-branch combination network that leverages lesion segmentation information for DL model training, focusing on the lesion area while simultaneously performing lesion segmentation and COVID-19 prediction^20^. As such, most studies have designed a severity assessment model utilizing either lung-masked or lesion-masked CT images^21^. To our knowledge, no study has yet developed a severity assessment model using both types of masked CT images and combined the DL model with quantitative features obtained from the lung area.

Consequently, this study introduces a hybrid approach that combines two DL models and one ML model, trained using quantitative features. Notably, the proposed model underwent rigorous external validation that included not only COVID-19 patients but also other types of IRDs including influenza, novel influenza, and BP.

## Methods

### Patient population

**Figure 1:**
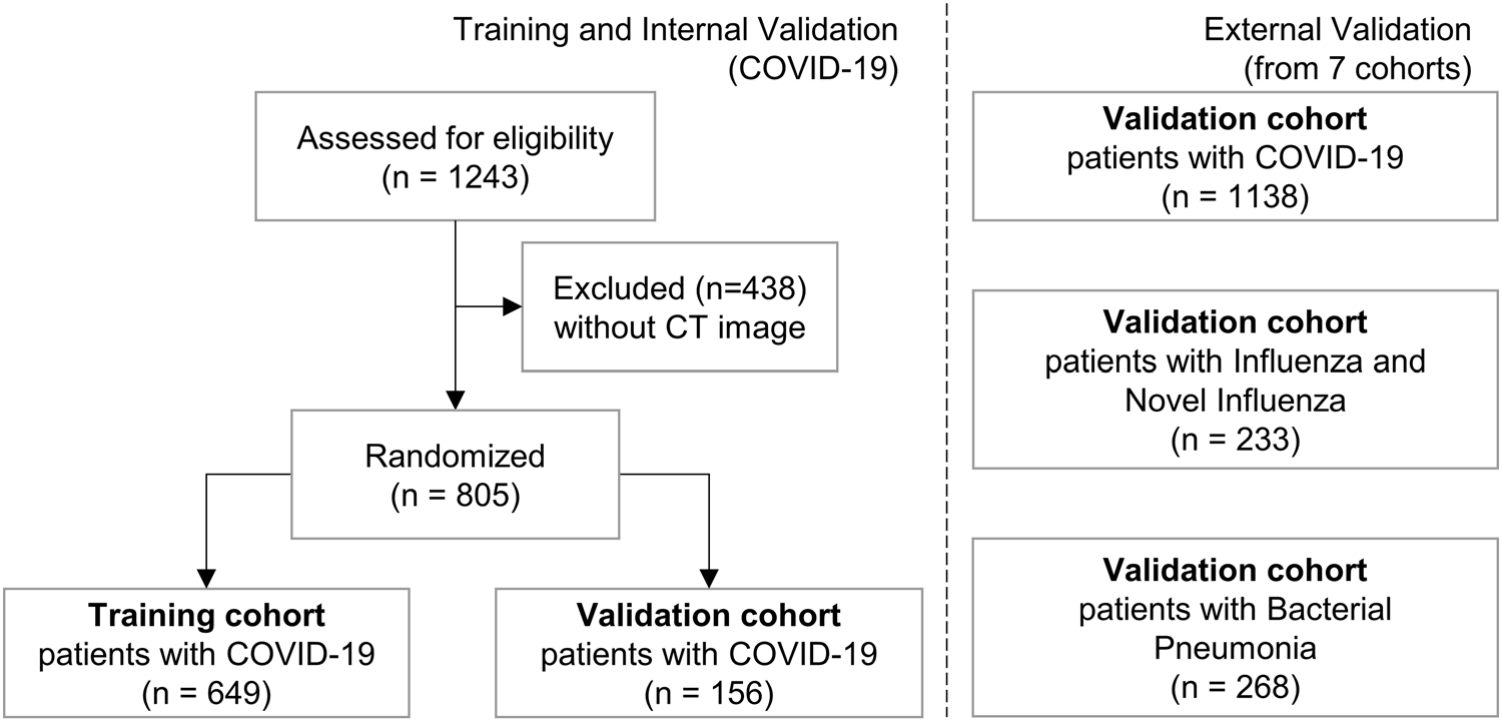
Data flow diagram of patients. COVID-19: coronavirus disease 2019. CT: computed tomography.

**Figure 2:**
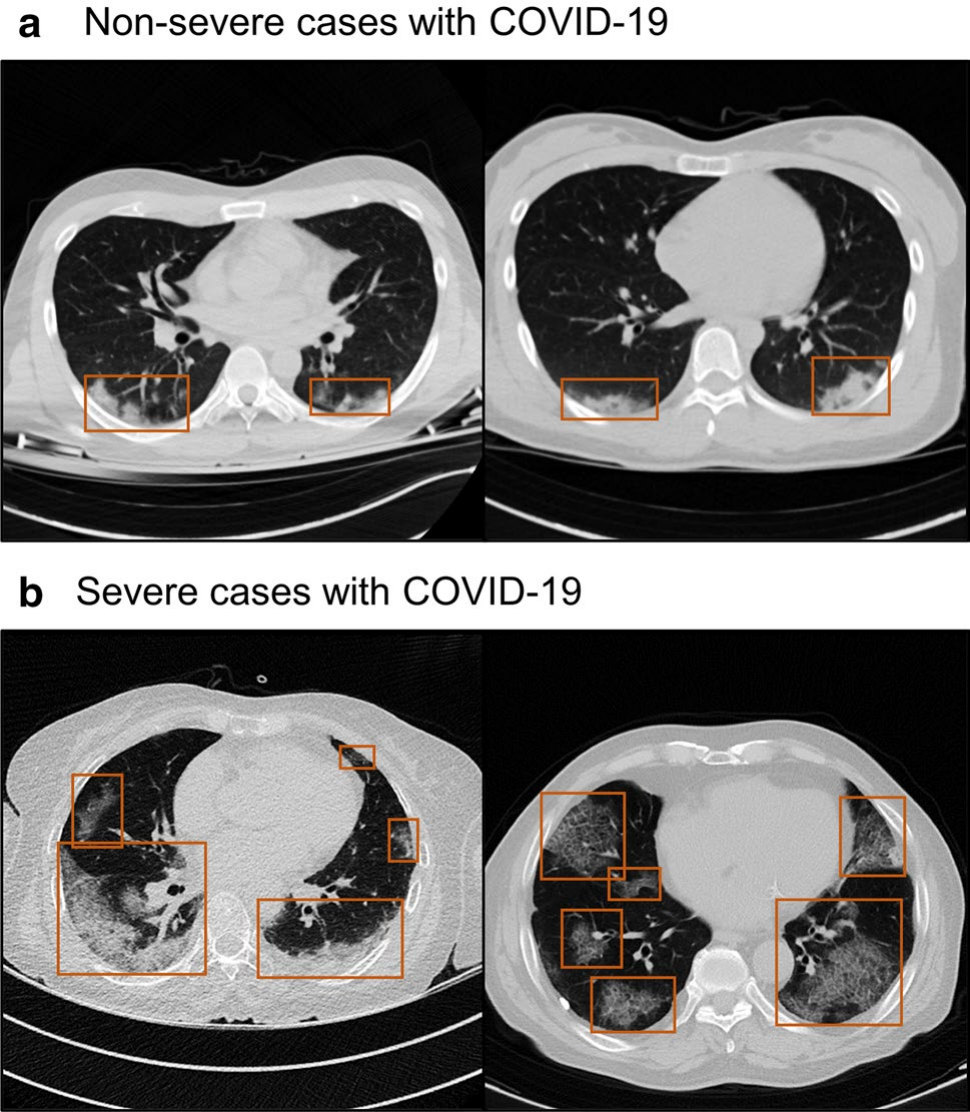
Example CT images with COVID-19. (**a**) Non-severe cases. (**b**) Severe cases. COVID-19, coronavirus disease 2019.

In this study, all research was performed in accordance with relevant guidelines/regulations and all experimental protocols were approved by the Institutional Review Board of Samsung Medical Center, Pusan National University Hospital, Chonnam National University Hospital, Keimyung University Dongsan Medical Center, Chungnam National University Hospital, Gachon University Gil Medical Center, Kyungpook National University Hospital, and Chungnam National University Sejong Hospital, which also waived written informed consent for this study. This study included data from 1243 patients diagnosed with COVID-19, admitted to a referral hospital. Data from 438 patients who did not undergo CT imaging were excluded from the analysis. The remaining 805 patients were randomly divided into two groups, allocating 80% of the participants for training and 20% for internal validation (referred to as D1). Three additional external data sets were collected from 7 different external cohorts. The external validation sets consisted of 1138 patients diagnosed with COVID-19 (referred to as D2), 233 patients with influenza and novel influenza (referred to as D3), and 268 patients with BP (referred to as D4), respectively. In this study, each patient was divided into severe and non-severe cases based on admission to the intensive care unit, mortality, and whether they received at least one of four specific treatments: steroid injection, oxygen supply, mechanical ventilation, or extracorporeal membrane oxygenation. A data flow diagram and the clinical characteristics of the patients are detailed in Fig. 1 and Table 1, respectively. Representative CT images from both non-severe and severe COVID-19 cases are provided in Fig. 2.

**Table 1:** Demographic and pathological characteristics.

Characteristics	No. (%) for training and internal validation			No. (%) for external validation					
	Training Set (n = 649)	Validation Set (n = 156) (D1)	*p* value*	Patients with COVID-19 (n = 1138) (D2)	*p* value*	Patients with influenza and novel influenza (n = 233) (D3)	*p* value*	Patients with bacterial pneumonia (n = 268) (D4)	*p* value*
Mean age (year)^†^	50 ± 17	52 ± 16	0.183	63 ± 16	< 0.001	67 ± 19	< 0.001	69 ± 18	< 0.001
Sex			0.976		0.395		0.015		< 0.001
Men	326 (50%)	78 (50%)		588 (52%)		93 (40%)		100 (37%)	
Women	333 (50%)	78 (50%)		550 (48%)		140 (60%)		168 (63%)	
Treatment									
Steroid injection	59 (9%)	11 (7%)	0.417	397 (35%)	< 0.001	19 (8%)	0.666	48 (18%)	< 0.001
Oxygen supply	21 (3%)	7 (4%)	0.444	288 (25%)	< 0.001	57 (24%)	< 0.001	110 (41%)	< 0.001
Mechanical ventilation	10 (2%)	2 (1%)	0.811	71 (6%)	< 0.001	8 (8%)	0.080	32 (12%)	< 0.001
ECMO	43 (7%)	7 (4%)	0.321	16 (1%)	< 0.001	1 (0%)	< 0.001	0 (0%)	< 0.001
Outcome									
ICU admission	140 (22%)	33 (21%)	0.909	62 (5%)	< 0.001	31 (13%)	0.006	44 (16%)	0.077
Mortality	143 (22%)	38 (24%)	0.533	62 (5%)	< 0.001	10 (4%)	< 0.001	29 (11%)	< 0.001
Severity			0.651		< 0.001		0.448		< 0.001
Non-severe	426 (66%)	106 (68%)		626 (55%)		160 (69%)		136 (51%)	
Severe	223 (34%)	50 (32%)		512 (45%)		73 (31%)		132 (49%)	

COVID-19, coronavirus disease 2019; ECMO, Extracorporeal membrane oxygenation; ICU, intensive care unit.

*p-values were calculated by comparing with the training set using the t-test or the chi-squared test.

^†^Mean ± standard deviation.

### CT acquisition parameters

The CT acquisition parameters for the dataset from one referral hospital were as follows: 34–782 mAs, 100–150 kVp, 0.625–5.00 mm slice thickness, and 0.35–0.97 mm pixel size. For the external validation sets, the CT acquisition parameters were unavailable due to data anonymization.

### DL model—segmentation

We employed the lung segmentation DL model from a previous study^22^ based on 2D U-Net^23^ (${\mathbf{U}\mathbf{N}\mathbf{e}\mathbf{t}}_{\mathbf{l}\mathbf{u}\mathbf{n}\mathbf{g}}$) and the VUNO Med-LungQuant (Seoul, Republic of Korea) version 1.0.0.4. for lesion segmentation based on 2D U-Net (${\mathbf{U}\mathbf{N}\mathbf{e}\mathbf{t}}_{\mathbf{l}\mathbf{e}\mathbf{s}\mathbf{i}\mathbf{o}\mathbf{n}}$). The input for the ${\mathbf{U}\mathbf{N}\mathbf{e}\mathbf{t}}_{\mathbf{l}\mathbf{u}\mathbf{n}\mathbf{g}}$ was axial CT images resized to 256 × 256 × 160, and the output was segmentation masks of the left and right lung, respectively, with a size of 256 × 256 × 160. The input for the ${\mathbf{U}\mathbf{N}\mathbf{e}\mathbf{t}}_{\mathbf{l}\mathbf{e}\mathbf{s}\mathbf{i}\mathbf{o}\mathbf{n}}$ was lung-masked CT images, and the output was segmentation masks of GGO and consolidation, respectively. The size of the input and output for the ${\mathbf{U}\mathbf{N}\mathbf{e}\mathbf{t}}_{\mathbf{l}\mathbf{e}\mathbf{s}\mathbf{i}\mathbf{o}\mathbf{n}}$ was 256 × 256 × 160.

### DL model—classification

**Figure 3:**
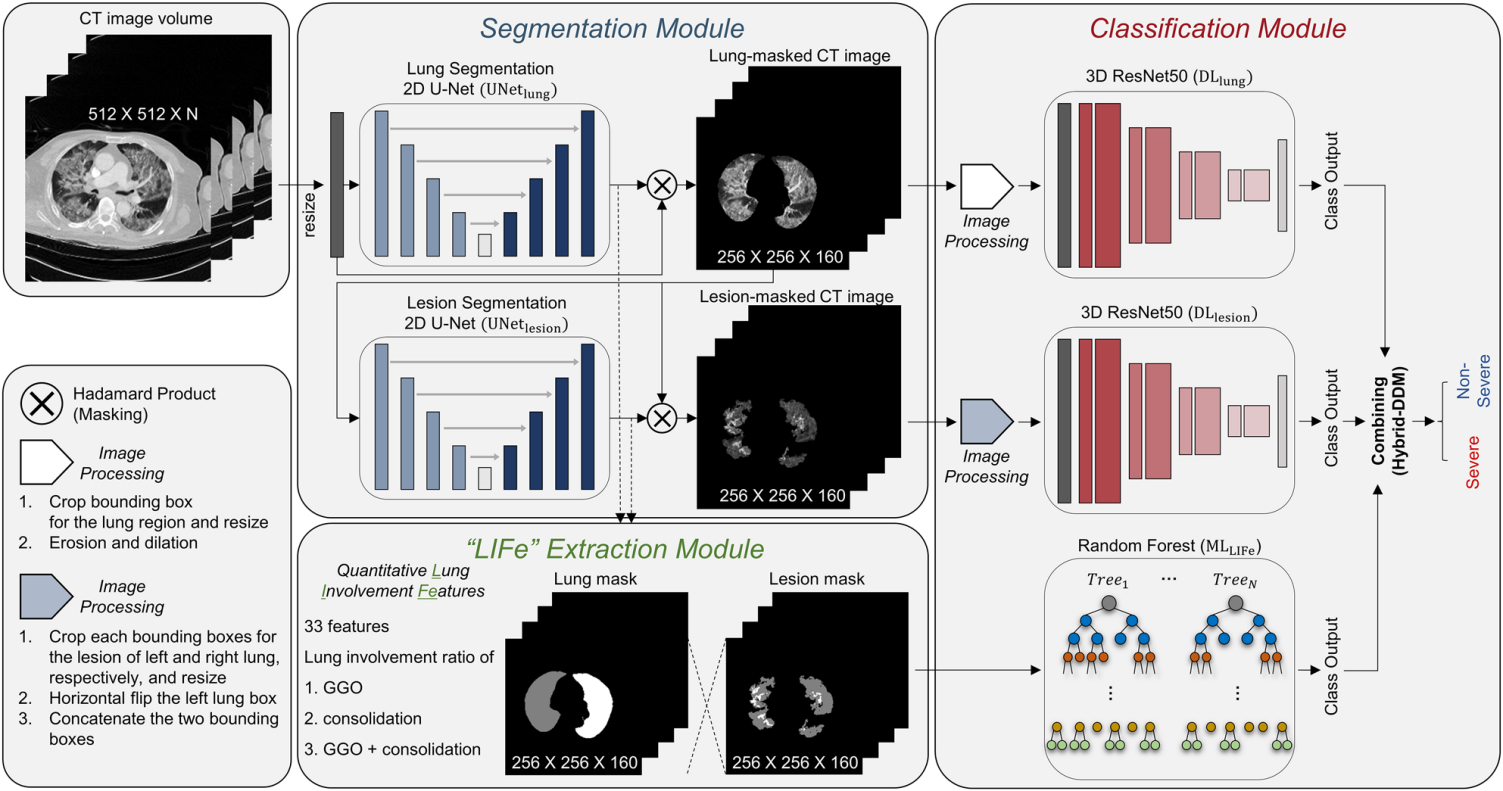
The overall flow of our proposed Hybrid-DDM model. CT, computed tomography. GGO, ground-glass opacity.

Two DL models were constructed based on 3D ResNet-50^24^ for the severity assessment. More details for ResNet-50 are available in Supplementary Fig. S1 online. 1) One DL model was trained with lung-masked CT images (${\mathbf{D}\mathbf{L}}_{\mathbf{l}\mathbf{u}\mathbf{n}\mathbf{g}}$). 2) The other DL model was trained with lesion-masked CT images (${\mathbf{D}\mathbf{L}}_{\mathbf{l}\mathbf{e}\mathbf{s}\mathbf{i}\mathbf{o}\mathbf{n}}$). The overall architecture of the proposed DL model is presented in Fig. 3. input for ${\mathbf{D}\mathbf{L}}_{\mathbf{l}\mathbf{u}\mathbf{n}\mathbf{g}}$ was the lung-masked CT image of size 192 × 192 × 160 obtained by cropping, masking, and resizing the segmented lung region, which was obtained by the elementwise multiplication of the CT image and the output of ${\mathbf{U}\mathbf{N}\mathbf{e}\mathbf{t}}_{\mathbf{l}\mathbf{u}\mathbf{n}\mathbf{g}}$. The output of ${\mathbf{D}\mathbf{L}}_{\mathbf{l}\mathbf{u}\mathbf{n}\mathbf{g}}$ was an estimate of the severity. The input for ${\mathbf{D}\mathbf{L}}_{\mathbf{l}\mathbf{e}\mathbf{s}\mathbf{i}\mathbf{o}\mathbf{n}}$ was the lesion-masked CT image of size 192 × 96 × 160 obtained by cropping, masking, and resizing the segmented lesion region, which was obtained by the elementwise multiplication of the CT image and the output of ${\mathbf{U}\mathbf{N}\mathbf{e}\mathbf{t}}_{\mathbf{l}\mathbf{e}\mathbf{s}\mathbf{i}\mathbf{o}\mathbf{n}}$. The output of ${\mathbf{D}\mathbf{L}}_{\mathbf{l}\mathbf{e}\mathbf{s}\mathbf{i}\mathbf{o}\mathbf{n}}$ was an estimate of the severity. Based on the same DL architecture, we constructed another DL model trained with lung-cropped CT images (baseline model) to analyze the effectiveness of lung or lesion masking. The input for the baseline model was the lung-cropped CT image of size 192 × 192 × 160, obtained by cropping and resizing the segmented lung region from ${\mathbf{U}\mathbf{N}\mathbf{e}\mathbf{t}}_{\mathbf{l}\mathbf{u}\mathbf{n}\mathbf{g}}$. The output of the baseline model was an estimate of the severity. The DL models were implemented using the Python Pytorch library. They were trained using the Adam optimizer^25^ with a weight decay of 0.0005 and a learning rate of 0.0001 with a cosine annealing schedular^26^ for 200 epochs. The DL models were trained using an Inter Xeon E5-2630 central processing unit and an NVIDIA GeForce GTX TITAN X graphics processing unit with CUDA version 11.4. Augmentation with a random axial rotation of − 15 to 15 degrees was performed during training. The training set was separated into 80% training and 20% validation (not the same as D1) sets.

### ML model—classification

Anatomically, there are 18–20 bronchopulmonary segments. Abnormal findings such as GGO and consolidation in each segment have different clinical significance^27,28^. However, segmenting individual bronchopulmonary segments is more challenging than segmenting bilateral lungs. Therefore, we used ${\mathbf{U}\mathbf{N}\mathbf{e}\mathbf{t}}_{\mathbf{l}\mathbf{u}\mathbf{n}\mathbf{g}}$ to segment only bilateral lungs and divided them into 4 segments each, which are simplified bronchopulmonary segments. As a result, there are 11 lung areas: right upper, right upper-middle, right lower-middle, right lower, whole right, left upper, left upper-middle, left lower-middle, left lower, whole left, and whole lung. Furthermore, there are three types of lesions: GGO, consolidation, and both GGO and consolidation. In conclusion, a total of 33 features (number of lung areas × number of lesion types) are obtained by calculating the proportions of lesions in each lung area, as summarized in Supplementary Table S1. In this study, we defined this feature set as quantitative Lung Involvement Features (LIFe). Using the LIFe, we constructed a random forest (RF)^29^ model. In this study, we defined this RF model as ${\mathbf{M}\mathbf{L}}_{\mathbf{L}\mathbf{I}\mathbf{F}\mathbf{e}}$ because the RF is one of the ML algorithms.

### Combination of DL and ML models

In this study, we proposed a hybrid model called Hybrid-DDM by the combination of two DL models ($\mathbf{D}{\mathbf{L}}_{\mathbf{l}\mathbf{u}\mathbf{n}\mathbf{g}}$ and $\mathbf{D}{\mathbf{L}}_{\mathbf{l}\mathbf{e}\mathbf{s}\mathbf{i}\mathbf{o}\mathbf{n}}$) and one ML model (${\mathbf{M}\mathbf{L}}_{\mathbf{L}\mathbf{I}\mathbf{F}\mathbf{e}}$). The Hybrid-DDM was obtained by uniform averaging the estimates of the severity from the three models.

### Statistical analysis

We used the following software: RF model construction (MATLAB. (2019). 9.7.0.1190202 (R2019a). Natick, Massachusetts: The MathWorks Inc.), statistical analysis (R Core Team (2020). R: A language and environment for statistical computing. R Foundation for Statistical Computing, Vienna, Austria. URL https://www.R-project.org/.). The severity assessment performance was evaluated with the area under the receiver operating characteristic (ROC) curve (AUC), and Delong’s method^30^ was used to compare two AUC values. A *p*-value lower than 0.05 was considered statistically significant.

## Results

**Figure 4:**
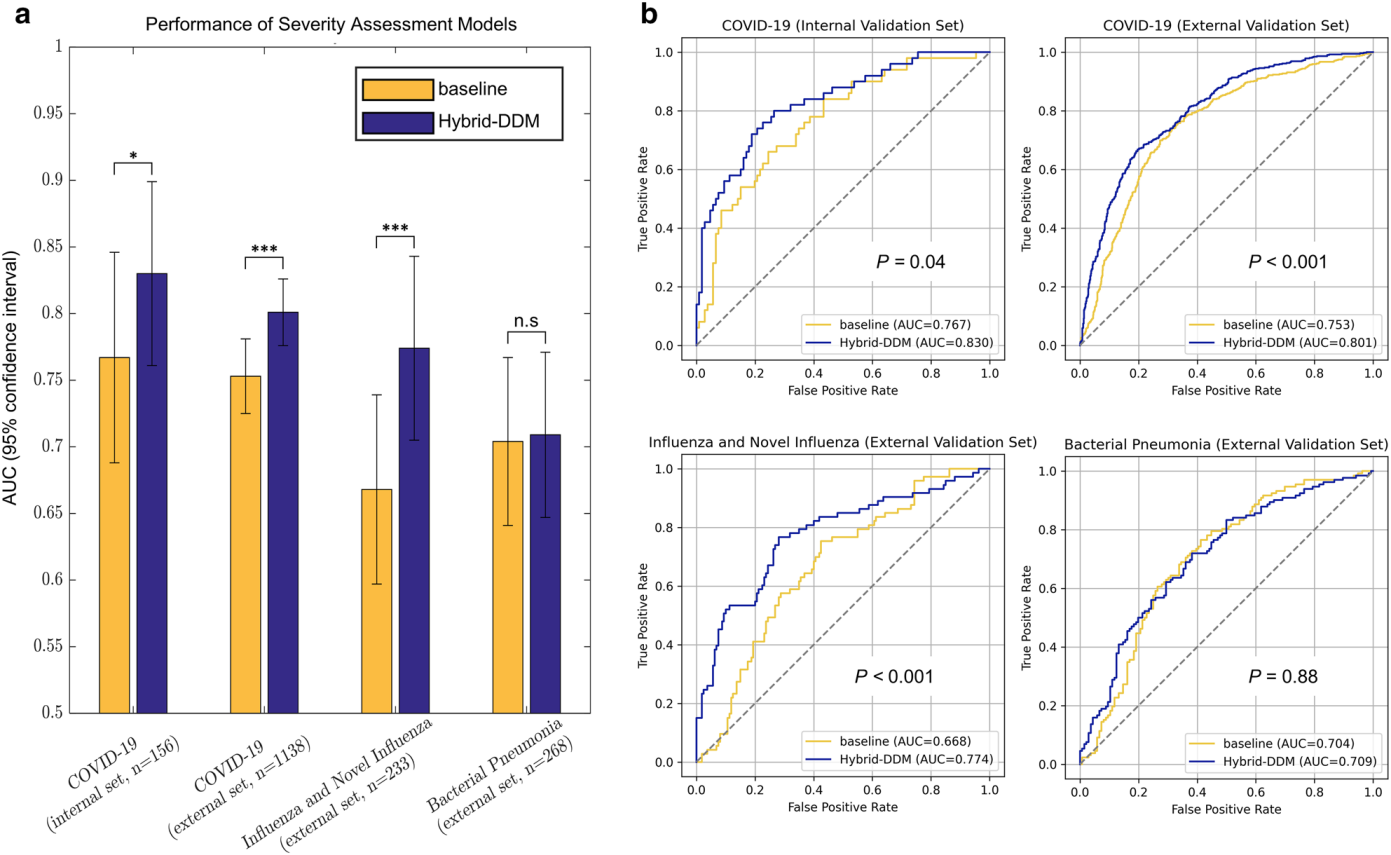
Performances of the severity assessment models. (**a**) Bar graph for model performance in each data set. (**b**) Receiver operating characteristic (ROC) curves of the severity assessment models. *: *p* < 0.05, **: *p* < 0.01, ***: *p* < 0.001. DL, deep learning. COVID-19, coronavirus disease 2019. AUC, area under the receiver operating characteristic curve.

The AUC and ROC curves of the Hybrid-DDM and the baseline models in each validation set are shown in Fig. 4a,b. More detailed results were summarized in Table 2.

**Table 2:** Performances of the severity assessment model for patients with COVID-19 and other three types of pneumonia.

Model	Internal validation				External validation											
	Patients with COVID-19 (*n* = 156) (D1)				Patients with COVID-19 (n = 1138) (D2)				Patients with influenza and novel influenza (n = 233) (D3)				Bacterial pneumonia (n = 268) (D4)			
	AUC (± 95% CI)	*p* value*	SEN	SPE	AUC (± 95% CI)	*p* value*	SEN	SPE	AUC (± 95% CI)	*p* value*	SEN	SPE	AUC (± 95% CI)	*p* value*	SEN	SPE
Baseline	0.767 (0.688–0.846)	*Reference*	0.660	0.755	0.753 (0.725–0.782)	*Reference*	0.740	0.687	0.668 (0.597–0.740)	*Reference*	0.753	0.575	0.704 (0.641–0.767)	*Reference*	0.765	0.588
${\mathrm{DL}}_{\mathrm{lung}}$	0.790 (0.711–0.869)	0.497	0.840	0.651	0.756 (0.727–0.785)	0.846	0.701	0.732	0.755 (0.686–0.824)	0.006	0.767	0.681	0.678 (0.614–0.742)	0.424	0.667	0.662
${\mathrm{DL}}_{\mathrm{lesion}}$	0.807 (0.728–0.887)	0.282	0.620	0.925	0.788 (0.761–0.814)	0.008	0.672	0.776	0.732 (0.657–0.808)	0.076	0.644	0.750	0.682 (0.618–0.745)	0.498	0.735	0.574
${\mathrm{DL}}_{\mathrm{lung}-\mathrm{lesion}}$	0.812 (0.735–0.888)	0.183	0.740	0.793	0.794 (0.768–0.820)	< 0.001	0.721	0.733	0.765 (0.696–0.834)	0.001	0.808	0.638	0.710 (0.648–0.772)	0.865	0.599	0.713
${\mathrm{ML}}_{\mathrm{LIFe}}$	0.811 (0.737–0.885)	0.244	0.640	0.868	0.777 (0.750–0.804)	0.066	0.734	0.701	0.754 (0.683–0.825)	0.017	0.575	0.850	0.698 (0.636–0.761)	0.844	0.727	0.640
Hybrid-DDM	0.830 (0.758–0.898)	0.036	0.800	0.736	0.801 (0.776–0.827)	< 0.001	0.668	0.804	0.774 (0.705–0.842)	< 0.001	0.767	0.719	0.709 (0.647–0.771)	0.877	0.720	0.618

Cut-off points for sensitivity and specificity were determined by the validation data of the training set (not the same as D1).

COVID-19, coronavirus disease 2019; AUC, area under the receiver operating curve; CI, confidence interval; SEN, sensitivity; SPE, specificity.

*p-values were calculated by Delong’s method.

The baseline model showed the lowest performances in D1, D2, and D3 with AUCs (95% confidence interval [CI]) of 0.767 (0.688–0.846), 0.753 (0.725–0.782), and 0.668 (0.597–0.740), respectively. The combination of the ${\mathbf{D}\mathbf{L}}_{\mathbf{l}\mathbf{u}\mathbf{n}\mathbf{g}}$ and the ${\mathbf{D}\mathbf{L}}_{\mathbf{l}\mathbf{e}\mathbf{s}\mathbf{i}\mathbf{o}\mathbf{n}}$ (${\mathbf{D}\mathbf{L}}_{\mathbf{l}\mathbf{u}\mathbf{n}\mathbf{g}-\mathbf{l}\mathbf{e}\mathbf{s}\mathbf{i}\mathbf{o}\mathbf{n}}$) showed the improved performance than each DL model with AUCs (95% CI) of 0.812 (0.735–0.888) versus 0.790 (0.711–0.869) and 0.807 (0.728–0.887) in D1, 0.794 (0.768–0.820) versus 0.756 (0.727–0.785) and 0.788 (0.761–0.814) in D2, and 0.765 (0.696–0.834) versus 0.755 (0.686–0.824) and 0.732 (0.657–0.808) in D3, respectively.

**Figure 5:**
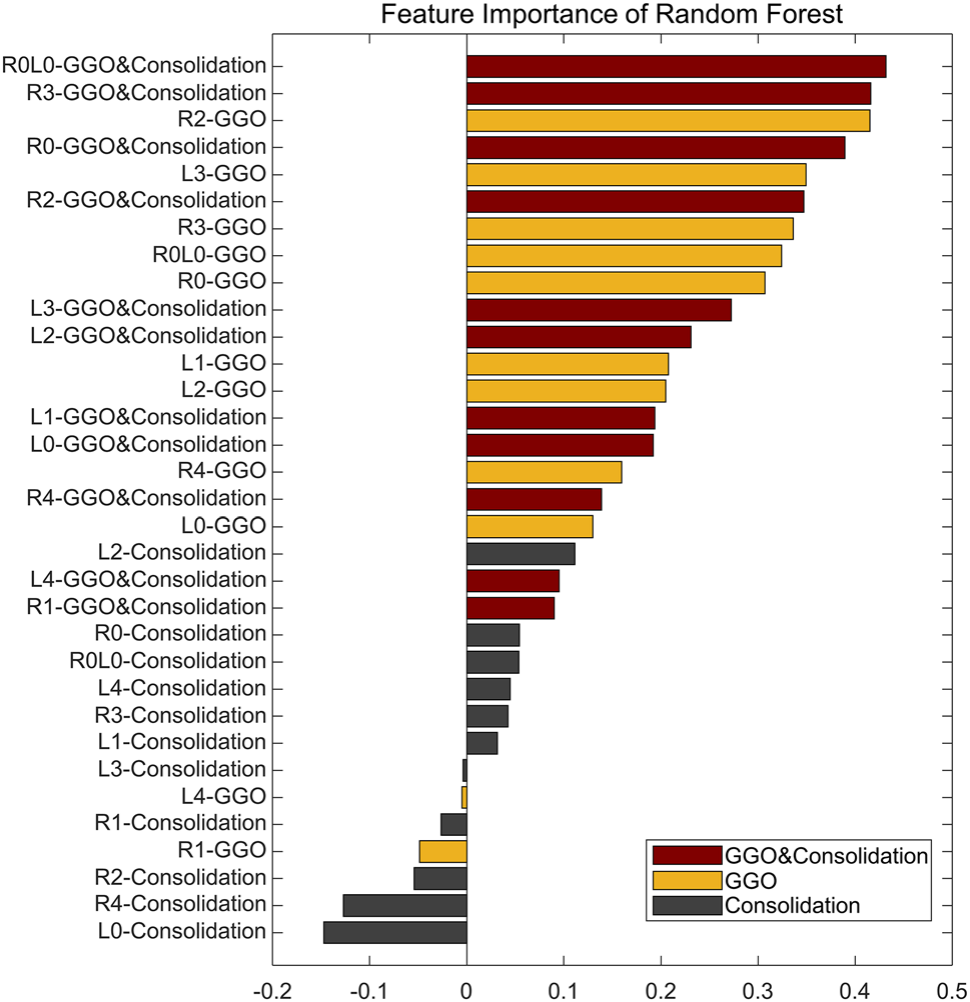
Feature importance of the random forest model (${\mathbf{M}\mathbf{L}}_{\mathbf{L}\mathbf{I}\mathbf{F}\mathbf{e}}$). A total of 33 features were used for model training.

**Figure 6:**
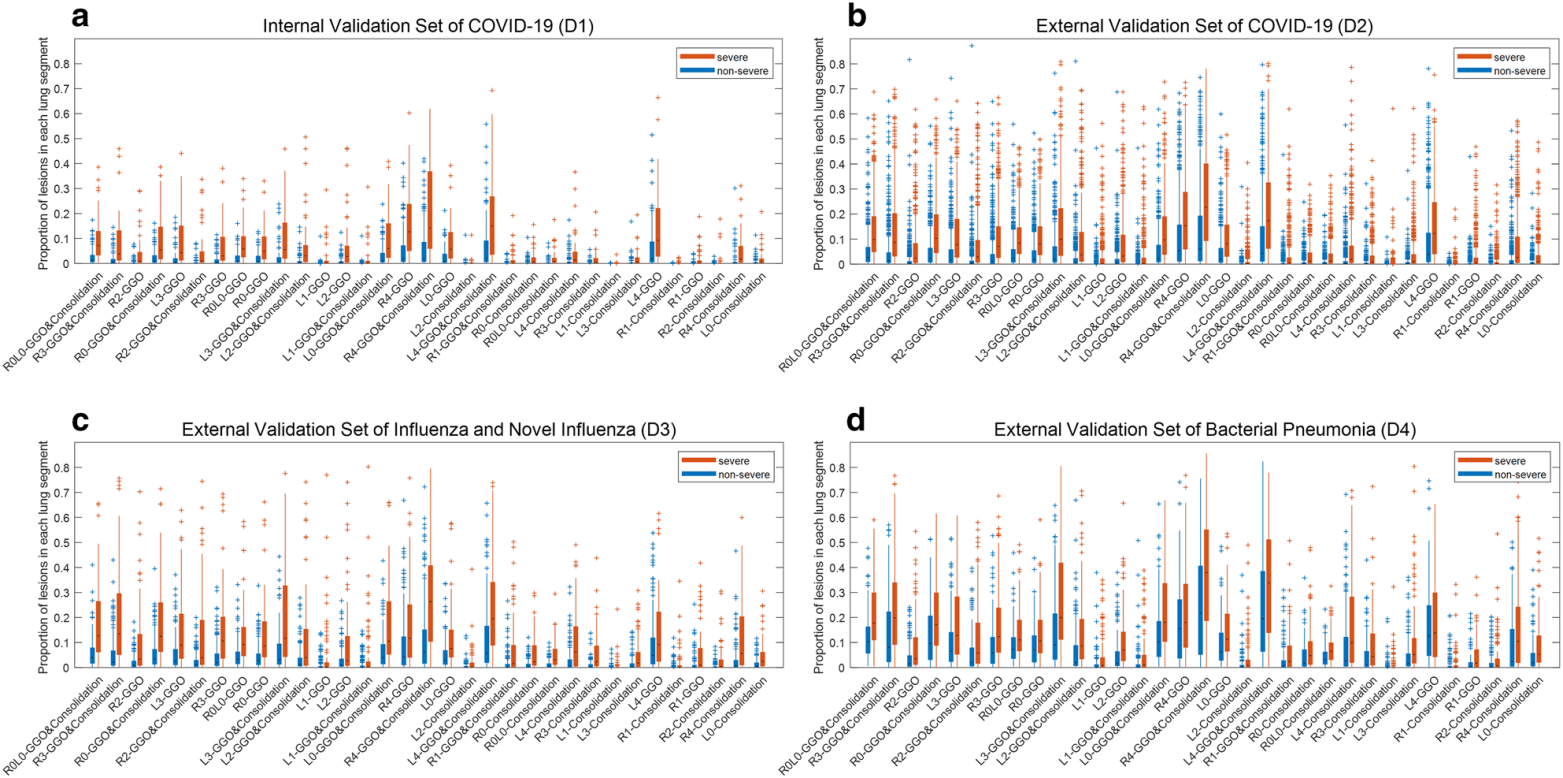
Visualization of the LIFe for severe and non-severe patients as a box plot. The LIFe is listed in descending order of feature importance from left to right. (**a**) Internal validation set of patients with COVID-19 (n = 156). (**b**) External validation set of patients with COVID-19 (n = 1138). (**c**) External validation set of patients with influenza and novel influenza (n = 233). (**d**) External validation set of patients with bacterial pneumonia (n = 268).

The ${\mathbf{M}\mathbf{L}}_{\mathbf{L}\mathbf{I}\mathbf{F}\mathbf{e}}$ showed comparable performance to the ${\mathbf{D}\mathbf{L}}_{\mathbf{l}\mathbf{u}\mathbf{n}\mathbf{g}-\mathbf{l}\mathbf{e}\mathbf{s}\mathbf{i}\mathbf{o}\mathbf{n}}$ with AUCs of 0.811 (0.737–0.885), 0.777 (0.750–0.804), and 0.754 (0.683–0.825) in D1, D2, and D3, respectively. Figure 5 shows the feature importance of 33 features used in the ${\mathbf{M}\mathbf{L}}_{\mathbf{L}\mathbf{I}\mathbf{F}\mathbf{e}}$. Feature importance in the RF model measures the contribution of each feature to the model's predictions. It is quantified by the degree to which the model’s performance decreases when the values of a particular feature are randomly permuted, thereby disrupting the relationship between the feature and the label. Consequently, a feature is considered highly important if its random permutation leads to a significant decline in the model's performance, indicating that the model relies heavily on this feature for accurate predictions^29^. As a result, GGO-related features tended to have higher importance than consolidation-related features. Box plots of 33 features for severe and non-severe patients are shown in Fig. 6. The features are listed in descending order of feature importance from left to right. In all datasets, severe patients tend to have larger feature values (the proportion of lesions) than non-severe patients. There is also a tendency for feature values to be larger in the lower lobes (L3, L4, R3, and R4) than in the upper lobes (L1, L2, R1, and R2).

Lastly, the Hybrid-DDM showed the highest performance in D1, D2, and D3 with AUCs (95% CI, *p*-value compared with baseline model) of 0.830 (0.758–0.898, *p* = 0.036), 0.801 (0.776–0.827, *p* < 0.001.), and 0.774 (0.705–0.842, *p* < 0.001.). However, in the D4, which is a set of BP patients, there was no significant difference in AUCs (95% CI) between the baseline model and the Hybrid-DDM (0.704 [0.641–0.767] vs 0.709 [0.647–0.771], *p* = 0.877).

## Discussion

This objective of this study was to develop an automated severity assessment model for patients with COVID-19 and other IRDs using CT images. To this end, we developed a Hybrid-DDM model that combined two DL models and one ML model. Our investigation yielded three crucial insights. Firstly, training a model with lung-masked or lesion-masked CT images enhanced the efficiency of severity assessment of patients with COVID-19, as compared to training solely with lung-cropped CT images. Secondly, the integration of two DL models with an ML model improved the performance of the severity assessment model. Thirdly, while the Hybrid-DDM model demonstrated significant effectiveness for patients with VP, it was not similarly effective for patients with BP.

The expedient severity assessment of patients with COVID-19 constituted a vital component of patient care and mandated immediate attention. Utilizing CT imaging for the diagnosis of disease severity in patients with IRDs provides clinicians with critical insights into the progression of the disease and potential responses to treatment, thereby enabling timely and tailored therapeutic interventions to enhance patient outcomes. Although the COVID-19 pandemic is currently in decline and transitioning into an endemic phase, it remains essential to prepare for future pandemics given their unpredictable nature. Consequently, it is crucial to establish reliable severity assessment systems for other IRDs. Numerous studies have risen to this challenge, exploring an array of techniques for severity assessment of patients with COVID-19 and other IRDs^31^. Specifically, many studies have adopted DL techniques that utilize lung or lesion information as guiding features during model training^20,32,33^. Our study aimed to assess the merits of employing differently pre-processed CT images for training DL models. Leveraging lung-masked CT images can be beneficial as it incorporates perilesional areas, which may encompass opacity, ambiguous lesions, and bronchus regions that are not discernible in lesion-masked CT images. Models trained on such comprehensive characteristics could estimate the severity of IRDs based on overall texture-based features instead of solely on lesion volume. On the contrary, the use of lesion-masked CT images allows the model to concentrate on lesion volume, texture, and shape, thus facilitating the determination of disease severity based on the presence and extent of lesions within the lung.

The combination of ${\mathbf{D}\mathbf{L}}_{\mathbf{l}\mathbf{u}\mathbf{n}\mathbf{g}}$ and ${\mathbf{D}\mathbf{L}}_{\mathbf{l}\mathbf{e}\mathbf{s}\mathbf{i}\mathbf{o}\mathbf{n}}$ demonstrated superior AUC compared to individual models for both the internal and external validation sets of patients with COVID-19 (0.812 vs 0.790 and 0.807 in D1, and 0.794 vs 0.756 and 0.788 in D2, respectively). This improvement was also evident in the external validation set of patients with influenza and novel influenza (0.765 vs 0.755 and 0.732 in D3), substantiating the effectiveness of combining ${\mathbf{D}\mathbf{L}}_{\mathbf{l}\mathbf{u}\mathbf{n}\mathbf{g}}$ and ${\mathbf{D}\mathbf{L}}_{\mathbf{l}\mathbf{e}\mathbf{s}\mathbf{i}\mathbf{o}\mathbf{n}}$ in severity assessment of patients with VP. Furthermore, the baseline model trained without any disease-related guidance was found to be inferior to both ${\mathbf{D}\mathbf{L}}_{\mathbf{l}\mathbf{u}\mathbf{n}\mathbf{g}}$ and ${\mathbf{D}\mathbf{L}}_{\mathbf{l}\mathbf{e}\mathbf{s}\mathbf{i}\mathbf{o}\mathbf{n}}$.

In clinical practice, the severity of COVID-19 and other IRDs can often be determined by evaluating the extent of lung involvement^15,16^. In order to quantitatively assess disease severity, we formulated a set of quantitative features, termed LIFe, which we subsequently utilized to construct a ML model. LIFe calculates the proportion of distinct lesion types, including GGO and consolidations. An added benefit of LIFe is that it eliminates the need for a lobe-specific segmentation mask, as it can be derived directly from the comprehensive lung segmentation mask. Our investigation showed that an ML model trained with LIFe features showed comparable performance to those of DL models. It is noteworthy that most lesions in patients with COVID-19 are typically located in the lower lobe of the lung^34,35^. Consistently, our study also found that LIFe values for patients with IRDs were higher in the lower lobe than in the upper lobe. In particular, the difference in GGO-related LIFe values between severe and non-severe patients was more marked than the difference in consolidation-related values. This insight guided us towards the development of a severity assessment model premised on measuring the proportion of lesions in bronchopulmonary segments, diverging from DL models that rely on nonlinear features extracted from the entire lung or specific lesions. However, a reliance solely on LIFe values might overlook other radiological features present in CT images. By incorporating DL models into the ML model, we were able to improve our severity assessment performance, as measured by the AUC, from 0.811 to 0.830 for D1, from 0.777 to 0.801 for D2, and from 0.754 to 0.774 for D3, respectively.

However, our approach did not prove effective for patients with BP. Even when incorporating prior information like lung masks, lesion masks, or LIFe values, the model's performance did not differ from DL models using lung-cropped CT images. This can be attributed to our training data being derived from patients with COVID-19, where one severe complication is VP^36^. Clinically, VP and BP exhibit distinct characteristics such as symptoms, disease severity, and radiological findings, which have been the subject of numerous studies^37,38^. In fact, we observed that the difference in LIFe values between severe and non-severe patients was smaller in BP patients than in VP patients. Thus, applying a severity assessment model trained on VP patients to BP patients would be unsuitable. Although our investigation featured a multi-institutional and multi-disease validation approach, the model fell short in enhancing performance for patients with BP. This underscores the imperative for cultivating more sophisticated and universally applicable methodologies that could be beneficial to patients with BP as well.

Our study had some limitations. Firstly, while our study includes data from multiple diseases, further validation should be performed on a variety of infectious respiratory diseases to ensure its applicability. Secondly, our research was conducted retrospectively, and prospective validation is required to strengthen its credibility. Thirdly, both internal and external datasets consist solely of patients from South Korea, so additional research is needed to extend its implications to other nations. Lastly, our model's practical utility could be improved if it were able to distinguish between VP and BP using CT images, laboratory findings, or clinicopathological information.

Despite these limitations, our findings have shown that training a DL model on CT images that were masked with either lung or lesion information, and subsequently integrating this with a ML model, significantly enhanced the performance of the severity assessment model for patients with VP. In the future, incorporating additional clinicopathological information such as age, gender, smoking history, or symptoms like cough, fever, and chills, could further improve the model's performance for the severity assessment of patients with IRDs.

### Supplementary Information

Supplementary Information.

## Data Availability

The datasets generated and/or analysed during the current study are not publicly available due to privacy issues but are available from the corresponding author on reasonable request.
