# Supplementary material for: Development and validation of a hybrid deep learning–machine learning approach for severity assessment of COVID-19 and other pneumonias
Source: Sci Rep. 2023 Aug 17;13:13420. doi: 10.1038/s41598-023-40506-w (PMC10435445; doi:10.1038/s41598-023-40506-w)
Supplement: Supplementary file 1 — Supplementary Information. [file 41598_2023_40506_MOESM1_ESM.docx]

# (Supplement) Development and Validation of A Hybrid Deep Learning-Machine Learning Approach for Severity Assessment of COVID-19 and Other Pneumonias

#
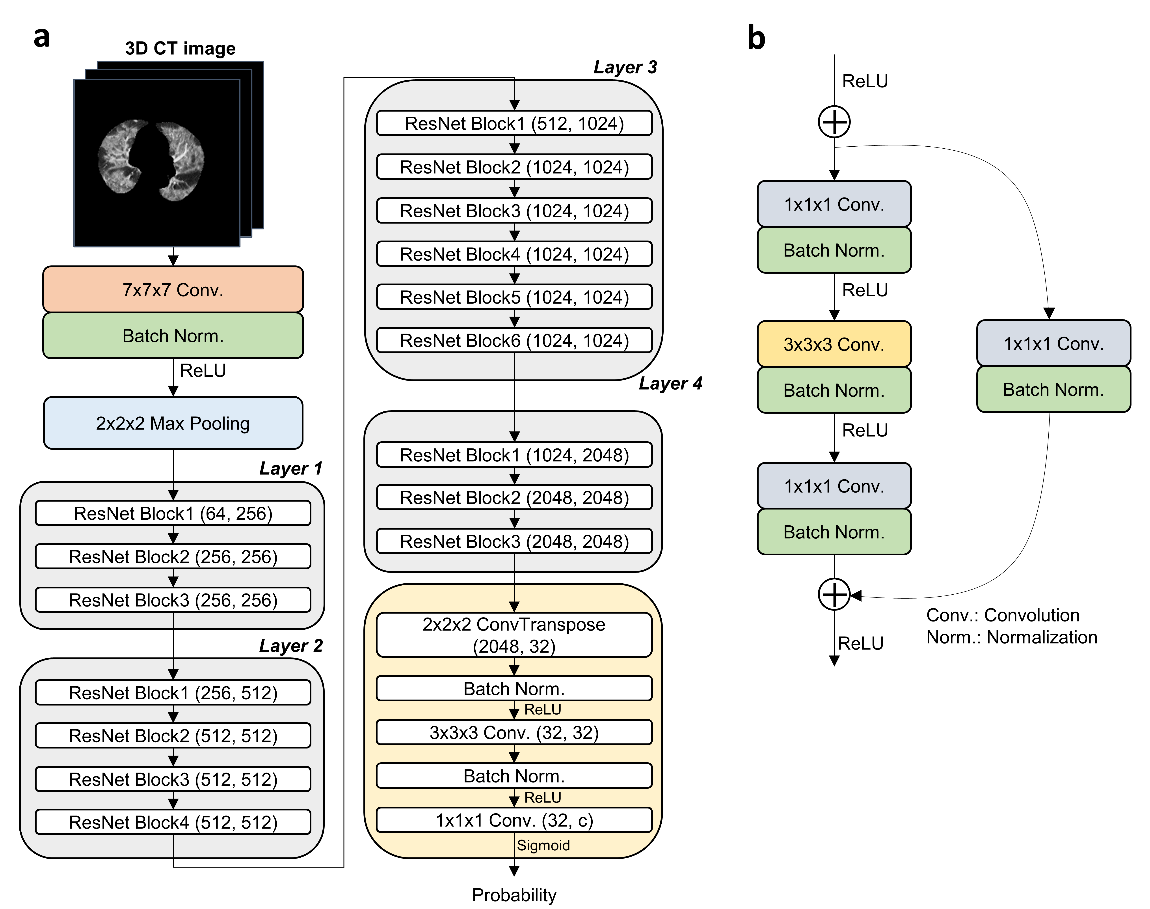


Figure S1. (a) Diagram of the 3D-ResNet50. (b) Diagram of the 3D ResNet Block.

| Features | CT Findings | Lung Segments (R: right, L: left, 0: whole, 1: upper, 2: upper middle, 3: lower middle, 4: lower) |
| --- | --- | --- |
| 1–8 | GGO | R1, R2, R3, R4, L1, L2, L3, L4 |
| 9–16 | Consolidation | R1, R2, R3, R4, L1, L2, L3, L4 |
| 17–24 | GGO + Consolidation | R1, R2, R3, R4, L1, L2, L3, L4 |
| 25–26 | GGO | R0, L0 |
| 27–28 | Consolidation | R0, L0 |
| 29–30 | GGO + Consolidation | R0, L0 |
| 31 | GGO | R0 + L0 (Bilateral Lung) |
| 32 | Consolidation | R0 + L0 (Bilateral Lung) |
| 33 | GGO + Consolidation | R0 + L0 (Bilateral Lung) |
| CT, computed tomography; GGO, ground-glass opacity | | |

Table S1. Extracted Quantitative Lung Involvement Features (LIFe)
